# Comparison of surgical resection of Axillary Lymph Nodes in Dogs with Mammary Gland Tumors with or without sentinel lymph node visualization with patent blue dye

**DOI:** 10.3389/fvets.2023.1149315

**Published:** 2023-05-12

**Authors:** Mayara Coutinho Carlo de Souza, Mayra Cunha Flecher, Fernanda Ming Arrais, Bruna Voltolin de Sena, Antonio Giuliano, Rodrigo dos Santos Horta

**Affiliations:** ^1^Department of Veterinary Clinic and Surgery, Universidade Estadual Paulista, São Paulo, Brazil; ^2^Departament of Veterinary Medicine, Universidade Vila Velha, Vila Velha, Espírito Santo, Brazil; ^3^Departament of Veterinary Medicine and Surgery, Veterinary School, Universidade Federal de Minas Gerais, Belo Horizonte, Minas Gerais, Brazil; ^4^Departament of Veterinary Clinical Science, Jockey Club College of Veterinary Medicine, City University of Hong Kong, Hong Kong, Hong Kong SAR, China

**Keywords:** female dogs, mammary gland neoplasm, lymphadenectomy, patent blue, prognosis

## Abstract

**Introduction:**

Dogs’ axillary lymph node (ALN) is often difficult to locate before surgical resection. The anatomical location of ALN often discourages Veterinarians from surgical lymphadenectomy. Considering the limited literature available, the actual incidence of metastases and the prognostic relevance are poorly understood.

**Methods:**

A non-randomized, prospective clinical study was conducted with female dogs (*n* = 41) with mammary gland tumor (MGT) in the thoracic or cranial abdominal mammary glands. The study investigated the risks of ALN metastasis based on tumors clinical findings, tumor size, histopathological diagnosis and grade. The main aim of this study was to compare ALN resection with or without patent blue 2.5% (PB) dye injection for sentinel lymph node visualization. A total of 46 mastectomies were performed and five animals underwent two mastectomies. In the first group, 17 patients underwent a mastectomy and lymphadenectomy without PB injection (G1). In contrast, in the second group, 24 patients also received PB injections for sentinel lymph node mapping (G2). The ALN was identified in 38/46 cases (82%). The ALN was identified and excised in only 58% of surgeries in G1(19/46), while in group 2, the lymph node was identified in 92% of the cases and resected in 100% of the cases. The use of PB improves ALN’s identification and reduces the surgical resection time in dogs with MGT.

**Results and discussion:**

Surgical time differed between the two groups, as it was significantly shorter in the PB injection group compared to group 1 (80 vs. 45 min) (*p* < 0.0001). The overall frequency of ALN metastasis was 32%. Macroscopic abnormalities in the lymph nodes, tumor size (>3 cm), and diagnosis of anaplastic carcinoma or grade II/III mammary gland tumors were associated with a higher probability of ALN metastasis. Metastases in the ALNs are more common, in dogs presenting with tumors larger than 3 cm and diagnosed with aggressive histological subtypes. The ALNs should be removed for correct staging, prognostic evaluation, and decision for adjuvant therapy.

## 1. Introduction

Mammary gland tumor (MGT) is one of the most common neoplasms in both women and intact female dogs. The biological behavior, diagnosis, treatment and prevention of canine MGT have been already extensively studied (1, 2). The similarities of women’s MGT with the canine counterpart include epidemiology, clinical, biological, histological and genetic similarities. Female dogs are considered an excellent comparative model for mammary tumors in women (3).

Despite all the available information regarding histological and molecular aspects of canine MGT, there is still no consensus on when the regional lymph node should be removed (1, 4). Regional lymphadenectomy is recommended for inguinal mammary tumors with rapid growth and/or larger than 3 cm, however for the same type of masses/tumors located in the thoracic mammary glands, the ALN is rarely removed (1). Thoracic mammary glands (M1 and M2) usually drain to the regional ALN, while cranial abdominal mammary gland (M3) drains to ALN and superficial inguinal lymph node, which also receives the lymphatic flow from caudal abdominal and inguinal mammary glands (M4 and M5) (5). The regional lymph node is defined based on the anatomic location, while the sentinel lymph node (SLN) is the first lymph node to receive the lymphatic flow from a solid neoplasm (6). The regional draining lymph node, based on the anatomical location and presence or not of metastasis affecting the normal lymphatic flow, can be different from the actual SLN. Identification of the SLN usually requires special techniques for their detection, such as direct or indirect lymphography (7). Palpation is a poorly sensitive method for the detection of lymph node metastases. In one study of dogs with palpable solid tumors, up to 85% of regional metastatic lymph nodes could not be detected by palpation alone (8). Although its clinical relevance is unclear, early metastases may be present in the form of isolated cells (submicrometastases up to 0.2 mm) or micrometastases (0.2–2.0 mm), without palpably enlarged lymph nodes (9). Surgical resection of the SLN or at least the regional lymph node, allows the early diagnosis of metastases, resulting in a more accurate staging and prognosis, which may improve disease control and drive the decision for adjuvant therapy (9). Although canine MGTs are very common in countries where female dogs are not early spayed, surgeons often find it challenging to identify and resect ALN, possibly resulting in underdiagnosis of early metastases (10).

An easy, convenient, safe, and low-cost technique for intra-operative identification of SLNs consists of the use of vital dyes (11, 12). This technique shows high sensitivity and specificity and is primarily used in women.

This study aimed to compare ALN resection with or without the aid of a vital dye, in dogs with mammary tumors. The secondary aim was to correlate the detection of lymph node metastasis to clinical findings, tumor size, histopathological diagnosis, and grade.

## 2. Materials and methods

This research was approved by the Ethics Committee on the Use of Animals from Universidade Vila Velha (Brazil), no. 377/2016, and all the owners signed informed consent to the study participation.

This prospective study included stage I–IV female dogs (*n* = 41) who underwent regional or chain mastectomy (*n* = 46) for treatment of MGT in the thoracic mammary glands and/or cranial abdominal mammary glands with ALN resection. Of the 41 female dogs, 5 underwent two mastectomies. In patients with tumors in M3, the superficial inguinal lymph node was also resected en bloc along with mastectomy. Patients with distant metastasis (stage V) were excluded. Complete staging included abdominal ultrasound and three views of thoracic radiography. Electrocardiogram, complete blood count and renal and hepatic serum biochemistry were also performed for the general health profile and the anaesthetic risk assessment. The same trained surgeon performed all surgical procedures.

All patients underwent general anesthesia. Morphine was administered for pre-anesthetic. Anesthesia induction was performed with propofol (4 mg/kg IV). The anesthetic was maintained with fentanyl CRI (5 μg/kg/h, IV) and isoflurane. Cefazolin (22 mg/kg, IV) was used 30 min prior to starting and meloxicam (0.2 mg/kg, IV) was given prior to induction.

In the first group, ALN resection was attempted in nineteen mastectomies (G1; *n* = 17) without vital dyes and surgery was performed with the standard technique in the author’s institution. Once the number of dogs in G1 was satisfactory for statistical analysis, the remaining dogs were included in group 2. In the second group, direct lymphography was used pre-surgically in twenty-seven mastectomies (G2; *n* = 24). For the direct lymphography, 0.5 ml of patent blue violet 2.5% (PB) was administrated by subpapillary injection, in the cranial or caudal thoracic mammary gland, ipsilateral to the tumor. The mammary papilla (nipple) was elevated with the digits and the dye was injected with a 26G needle. A waiting time of 10 min was taken to allow the dye to drain and pigment to the SLN. Shortly lymphadenectomy was performed soon after, followed by a mastectomy.

For ALN resection an oblique skin incision was performed in the axillary region at the level of first and second ribs. After the identification of the ALN, ligation of the vein, artery and lymphatic vessel was performed en bloc with 3-0 polyglactin 910 (Shalon^®^). The inguinal lymph node was removed in cases of complete unilateral chain mastectomy (mammary strip), or when associated with contralateral M5 removal.

The surgical technique involved the removal of the tumor (s), with lateral safety margins of 2–3 cm and one deep fascial plane. The decision on the surgical approach was based on the clinician’s preference based on stage of the patient, location, number and size of lesions using a caliper, as well as the presence of adhesions, necrosis or ulceration. The time of surgery was monitored and recorded from the start of the incision to the end of the last suture.

After resection, surgical specimens were stored in 10% formalin and sent for histopathological examination. The ALN was identified and sent in a separate container and the superficial inguinal lymph node along with the mammary chain.

In the first 24 postoperative hours, the patients were kept hospitalized for pain control with morphine^1^ (0.5 mg/kg q4h IM) and dipyrone^2^ (25 mg/kg q8h IV). If the patient was uncomfortable, morphine at 0.25 mg/kg, IM was given as an additional analgesic. After hospital discharge, dogs were sent home with a prescription of oral meloxicam (0.1 mg/kg, every 24 h), tramadol hydrochloride (6 mg/kg, every 8) and dipyrone (25 mg/kg, every 8 h), all for five days. If pre-operative mammary gland infection was present, Cefazolin^3^ (20 mg/kg q8h IV) was given in the hospital, and then the patient was discharged with oral Cephalexin (22–30 mg/kg, q 12 h) for ten days.

Histopathological classification of mammary neoplasms was performed according to Goldschmidt et al. (13) and Cassali et al. (1) and graded accordingly to the Nottingham System (1). The lymph nodes were sectioned according to the sashimi method, with cuts of 2 mm with the maintenance of perinodal adipose tissue to include the entire lymph node (14).

### 2.1. Statistical analysis

Statistical analysis was performed with Graph Pad Prism v. 6.02. The variables were tested for homogeneity and normality through the Kolmogorov–Smirnov test. Differences were considered significant if *p* < 0.05 (15).

The experimental design divided the patients into two groups: G1 ALN resection without the lymphography and G2, with the PB lymphography. The tumor (s) and regional lymph node (s) were classified based on clinical (tumor’s size, adhesion and ulceration, lymph node’s shape, consistency, and volume) and histopathological findings (tumor’s classification and grade, lymph node’s presence of metastases) (15).

To evaluate the homogeneity between the two groups, the mean age of the patients was compared by the student’s *t*-test (normal distribution), and their weight by the Mann–Whitney test (non-normal distribution). The sensitivity and specificity of the SLN biopsy technique were analyzed based on the percentage of the absence of staining to other tissues, other than lymph node/lymphatic vessels, and the correct lymph node staining. The time of ALN dissection in both groups was estimated and compared using the Mann–Whitney test (non-normal distribution). The chi-square test was used to evaluate the difference in the frequency of distribution of macroscopic abnormalities in the lymph nodes (between groups and in the presence or absence of lymph node metastasis), metastases in axillary and inguinal lymph nodes (between groups), tumors clinical findings (tumor ulceration/necrosis/adhesion to underlining structures), tumor size, histopathological diagnosis and grade (between groups, mammary glands and in the presence or absence of lymph node’s metastasis) (15).

## 3. Results

Of the 41 female dogs included in this study, nine were mixed-breed (22%) and the remaining were distributed in the following breeds: Poodle (*n* = 13), Yorkshire Terrier (*n* = 4), Doberman Pinscher (*n* = 3), Lhasa Apso (*n* = 3), Shih Tzu (*n* = 1), Maltese (*n* = 1), Miniature Schnauzer (*n* = 1), Cocker Spaniel (*n* = 2), Labrador Retriever (*n* = 2), Rottweiler (*n* = 1), American Pit Bull Terrier (*n* = 1). Only nine female dogs were spayed (22%). Regarding the use of hormonal contraceptives (i.e., estradiol cypionate, medroxyprogesterone acetate, megestrol acetate), it was not possible to obtain this information in 16 cases, however, two female dogs used some contraceptive and 23 (56%) did not use it. Seven female dogs (17%) presented a history of pseudocyesis. The ages ranged from four to 15 years old (10.7 ± 2.8 years). The weight varied between 3 and 35 kg (10.8 ± 9.4 kg). Table 1 presents patients’ clinical data and surgical time in groups 1 and 2.

**Table 1:** Clinical characteristics and surgical time of female dogs with mammary gland tumors in groups 1 (without patent blue stain of axillary lymph nodes) and 2 (with patent blue).

		Group 1 (*n* = 17 female dogs)	Group 2 (*n* = 24 female dogs)	Total (*n* = 41 female dogs)	Statistics (*p*-value)
Age (years, mean ± standard deviation)		9.9 ± 3.3 (a)	10.9 ± 2.7 (a)	10.7 ± 2.8	Student “*t*” test (*p* = 0.3)
Weight (Kg)	Mean ± standard deviation	12.6 ± 10.2	9.7 ± 8.9	10.9 ± 9.4 kg	Mann–Whitney (*p* = 0.4)
	Median	7.4 (a)	6.3 (a)	6.8	
Surgical time (minutes)	Mean ± standard deviation	75.6 ± 17.8	45.5 ± 9.5	57.3 ± 19.8	Mann–Whitney (*p* < 0.0001)
	Median	80.0 (a)	45.0 (b)	50.0	

Different lowercase letters on the same line indicate statistically significant differences.

Since five dogs underwent two mastectomies, 46 surgeries were performed, including unilateral chain mastectomy (1–5) (*n* = 27); regional cranial mastectomy involving the thoracic mammary glands without the cranial abdominal (1–2) (*n* = 8); and unilateral chain mastectomy involving the contralateral caudal block (1–5 plus the 4–5 of the contralateral chain) (*n* = 11). Among the non-spayed female dogs (*n* = 29), 14 were spayed at the same event. The linea alba was closed before the regional cranial mastectomy (en-bloc removal of 1–2) was performed. Lumpectomies were performed in seven procedures in the contralateral chain.

ALN resection allowed the identification and excision of a single ALN in 38 of 46 surgical procedures (82.6%). In G1, it was possible to find the ALN and perform the excision in only 58% of the cases (11/19). In group 2, PB stain of the ALN was achieved in 92% of the patients (25/27) and identification of lymph node was possible in all cases (*p* = 0.005, chi-square test). Despite the lack of dye staining of the lymph node in 2 dogs in G2, those lymph nodes were also identified and removed (one of these dogs had an anaplastic carcinoma with lymphatic infiltration and lymph node metastasis) Surgical time was significantly different between G1 and G2 (median of 80 and 45 min, respectively) (Mann–Whitney, *p* < 0.0001) (Table 1).

**Figure 1:**
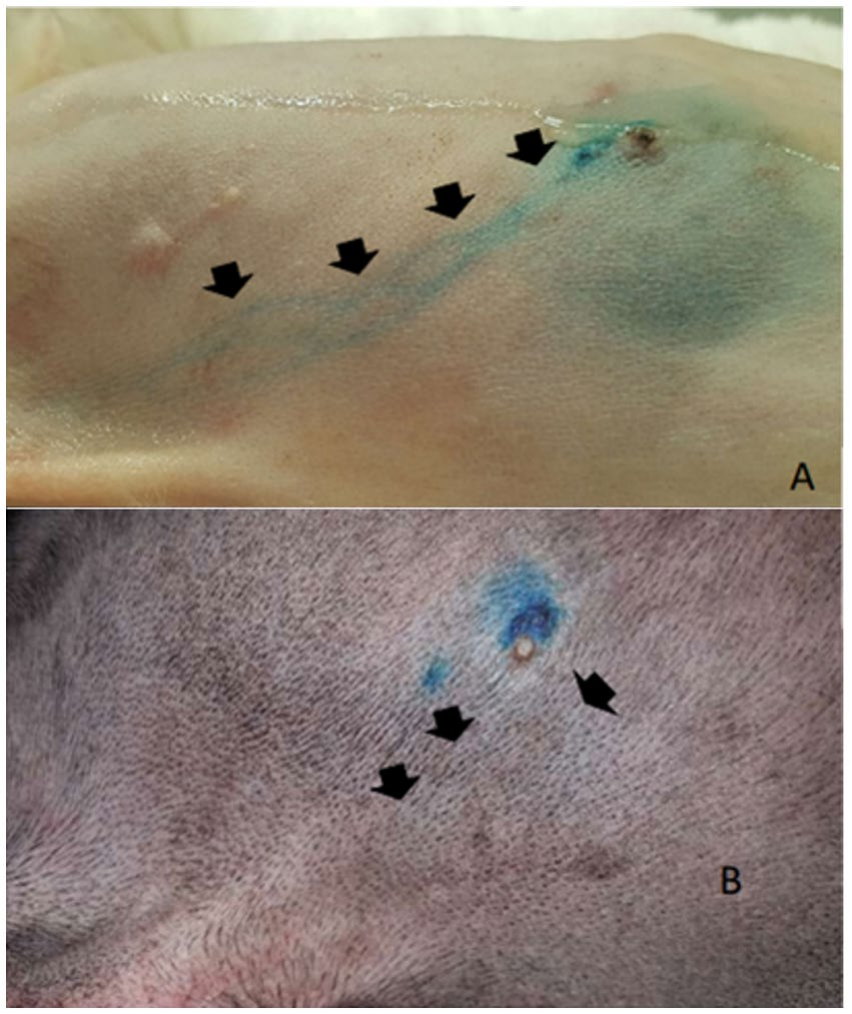
Subapillar administration of patent blue 2.5%. **(A)** Administration in the caudal thoracic mammary gland dye stands out on the skin in a light-skinned dog (arrows). **(B)** Administration in the cranial thoracic mammary gland with poor visualization of the regional drainage (arrows).

**Figure 2:**
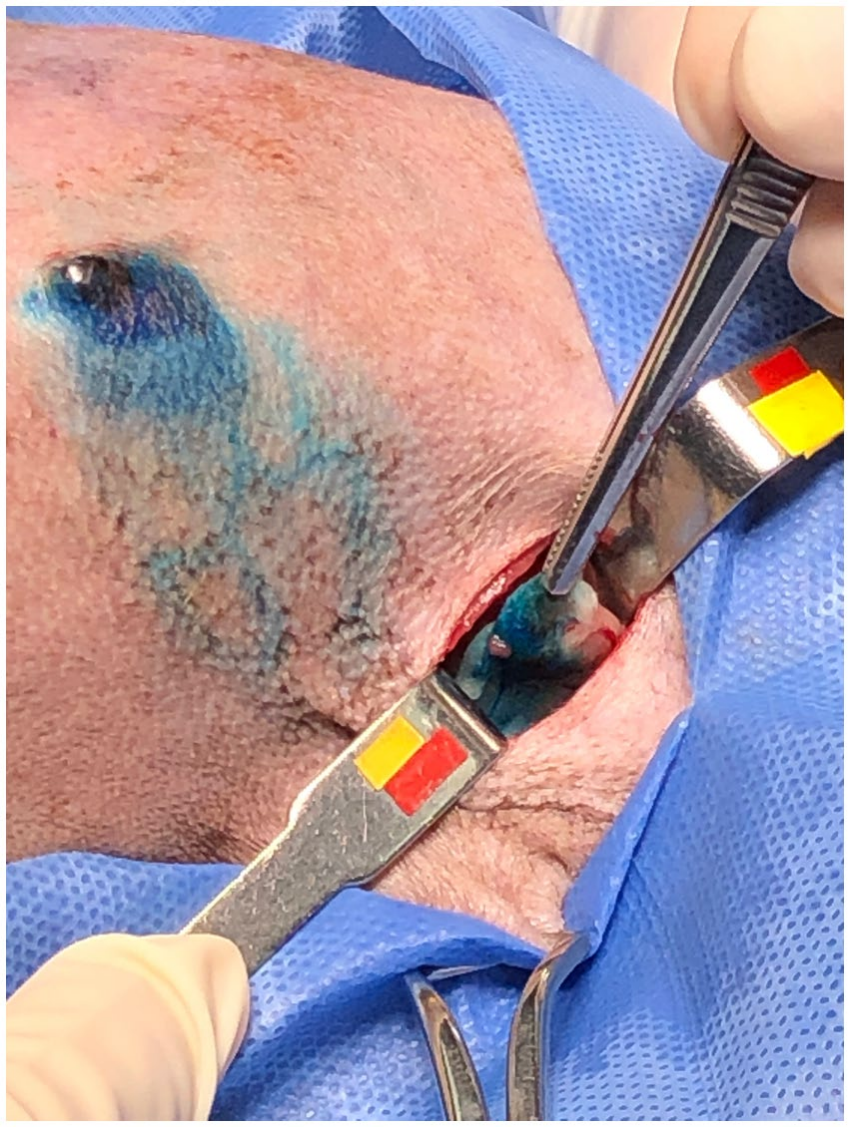
Female dog with the axillary lymph node stained with patent blue 2.5%.

**Figure 3:**
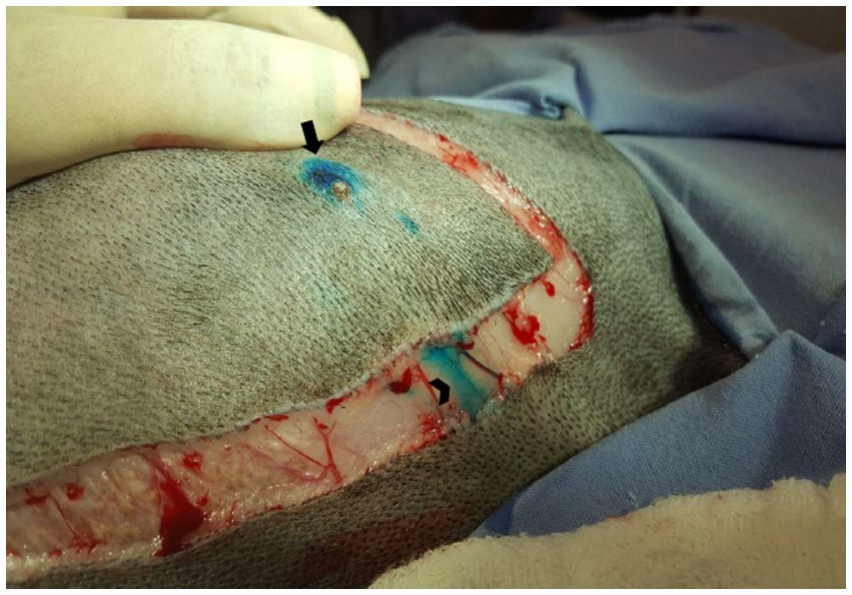
Female dog after administration of patent blue 2.5% and with skin incision. Subpapillary pathway (arrow) and lymphatic vessels draining the dye to the axillary region (arrowhead).

After the PB dye administration, it was possible to visualize the vital dye drainage after 5–10 min, especially in very small and old dogs with thin skin (Figures 1A,B). After approximately 10–15 min the ALN was already well stained (Figure 2) and easily identified. For its identification and removal, it was only necessary to follow the lymphatic vessels stained in blue (Figure 3). It was also observed that, after approximately 20 min from its administration, the dye begins to leave the lymphoid organ and the coloration becomes weaker. No allergic reactions to the PB were observed.

The removed neoplasms were categorized based on their size, adhesion, and ulceration. The dogs had at least one tumor and a maximum of seven; among these, only three dogs had only one nodule. Regarding the tumor size, 43% (55/126) presented between 1 cm and 3 cm, followed by 29% (37/126) of tumors larger than 5 cm, 16% (20/126) smaller than 1 cm and 11% (14/126) between 3 cm and 5 cm. The largest tumor observed was 20 cm. At least one tumor attached to the skin or musculature was found in 8/41 dogs (19%), completing a total of nine tumors with adherence. At least one ulcerated tumor was detected in 11/41 dogs (26.8%), with a total of 13 ulcerated tumors.

Histopathological examinations were performed separately for all removed tumors (*n* = 127, 126 of which were actual mammary neoplasms) and lymph nodes (*n* = 87, of which 38 were axillary and 49 were inguinal). A grade III mast cell tumor was diagnosed in the dermis, near the cranial thoracic mammary gland, in a dog with anaplastic carcinoma in the caudal thoracic mammary gland. Among the MGT (*n* = 126), 88 were malignant (69.8%), 22 benign (17.5%) and 16 non-neoplastic (12.7%). Animals with benign tumors or non-neoplastic lesion had at least one malignant tumor.

According to the classification of Cassali et al. (1), mixed mammary tumour was the most common malignancy (36.4%), while for Goldschmidt et al. (13) the histologically similar complex carcinoma was present in 22.7% of cases, followed by tubular carcinoma (12.5%), ductal carcinoma [or papillary ductal carcinoma according to (1)] and carcinosarcoma (each representing 6%), solid carcinoma and anaplastic carcinoma (each representing 4%). A case of micropapillary carcinoma (1.1%) was also found. Eighty-seven malignant tumors were graded accordingly to the Nottingham system as grade I (56.4%; 49/87), grade II (32.1%; 28/87) and grade III (11.5%; 10/87). It was not possible to grade an anaplastic carcinoma, due to the absence of tubular formation and low cellularity (predominance of necrotic tubules, lymphatic infiltration, and neoplastic emboli).

Differences in the variables examined including physical, tumor size, histopathological classification and grade according to the presence or absence of metastasis in the axillary and superficial inguinal lymph nodes are shown in Table 2. Metastases were identified in 8/49 inguinal lymph nodes (16.3%) and 12/38 ALNs (31.6%). It is important to note that from all the lymph nodes removed, only five presented macroscopic changes on physical examination, increased size, and consistency (four axillaries and one superficial inguinal). Four of these lymph nodes presented with metastasis (three axillaries and one superficial inguinal). Of all the confirmed metastatic lymph nodes only 20% (4/20) were abnormal at physical examination. Clinical macroscopic or palpable abnormalities in the lymph nodes (axillary or inguinal) were associated with a higher probability of metastasis, with *p* = 0.05 and 0.02, respectively (chi-square).

**Table 2:** Clinical features, histopathological diagnosis and grading for canine mammary gland tumors with or without nodal metastasis.

		Cassali et al. (1)	Goldschmidt et al. (13)	With metastasis			Without metastasis			Chi-square (*p*-value)
				Group 1	Group 2	Total	Group 1	Group 2	Total	
Axillary lymph node (*n* = 38)	Abnormalities in physical examination	Yes		2	1	3	1	0	1	***p*** = **0.05**
		No		5	4	9	7	18	25	
	Diagnosis in the thoracic mammary glands (+ cranial abdominal)	Ductal ectasia		0	0	0	2	1	3	–
		Lobular hyperplasia		0	0	0	0 (+1)	0	1	–
		Atypical Lobular Hyperplasia		0	0	0	1	1	2	–
		Complex adenoma		0	0	0	0 (+1)	0	1	–
		Tubular adenoma		0	0	0	2 (+2)	8 (+5)	17	–
		Benign mixed tumor		0	0	0	2 (+1)	2 (40%)	5	–
		Total (benign and non-neoplastic)		0	0	0	12	17	29	
		Carcinoma *in situ* in duct papilloma	Papillary cystic carcinoma	1 (100%)	0	1 (100%)	0	0	0	*p* = 0.1
		Mixed tumor carcinoma	Carcinoma complex	0 (+1)	0	1	1 (+1)	5 (+5)	12	
		Mixed tumor carcinoma		1 (5%)	2 (+3)	6	6	6 (+2)	14	*p* = 0.8
		Papillary ductal carcinoma	Ductal carcinoma	0	0	0	1 (25%)	1 (+2)	4	*p* = 0.2
		Tubular carcinoma		0	0	0	3 (+1)	1	5	*p* = 0.2
		Malignant adenomyoepithelioma		0	0	0	0	1	1	*p* = 0.5
		Solid carcinoma		1 (+1)	0	2	0	1	1	*p* = 0.1
		Carcinosarcoma		0	0	0	0	1	1	*p* = 0.5
		Carcinoma anaplastic		2	2	4	0	0	0	***p*** = **0.0008**
		Micropapillary carcinoma		0	0	0	0	0 (+1)	1	*p* = 0.5
		Squamous cell carcinoma		0	0 (+1)	1	0	0	0	*p* = 0.1
		Total (malignant)		7	8	15	12	26	38	–
	Grade	I (A)		1	0	1	10	13	23	***p*** = **0.004**
		II (B)		2	5	7	1	7	8	
		III (B)		4	0	4	0	5	5	
	Size	<1.0 cm (A)		1	0	1	5	5	10	***p*** = **0.02**
		1–3 cm (A)		1	0	1	11	19	30	
		3–5 cm (AB)		0	3	3	2	4	6	
		>5 cm (B)		5	2	7	2	6	8	
Inguinal lymph node (*n* = 49)	Abnormalities in physical examination	Yes		0	1	1	0	0	0	***p*** = **0.02**
		No		5	2	7	15	26	41	
	Diagnosis in the caudal abdominal and inguinal mammary glands (+ cranial abdominal)	Ductal ectasia		0	0	0	2	0		–
		Lobular hyperplasia		0	0	0	2 (+1)	0	3	–
		Atypical Lobular Hyperplasia		0	0	0	0	2	2	–
		Ductal papilloma		0	0	0	0	1	1	–
		Complex adenoma		0	0	0	1	1	2	–
		Tubular adenoma		0	0	0	2	1 (+2)	5	–
		Benign mixed tumor		0	0	0	1 (+1)	2	4	–
		Total (benign and non-neoplastic)		0	0	0	10	9	19	–
		Carcinoma *in situ* in duct papilloma	Papillary cystic carcinoma	0	0	0	1	0	1	*p* = 0.6
		Ductal carcinoma *in situ* comedo type	Cribriform carcinoma	0	0	0	1	0	1	*p* = 0.6
		Mixed tumor carcinoma	Carcinoma complex	1 (+1)	0	2	0 (+1)	6 (+5)	12	*p* = 0.6
		Mixed tumor carcinoma		1	3 (+1)	5	5	6 (+2)	13	*p* = 0.3
		Papillary carcinoma	Papillary tubule carcinoma	0	0	0	1	0	1	*p* = 0.6
		Papillary ductal carcinoma	Ductal carcinoma	0	0	0	0	1 (+2)	3	*p* = 0.4
		Tubular carcinoma		1	0	1	0	3	3	*p* = 0.8
		Solid carcinoma		0	0	0	0 (+1)	1	2	*p* = 0.5
		Carcinosarcoma		1	0	1	0	2 (+1)	3	*p* = 0.4
		Micropapillary carcinoma		0	0	0	0	0 (+1)	1	*p* = 0.6
		Squamous cell carcinoma		0 (+1)	0	1	0	0	0	*p* = 0.04
		Total (malignant)		6	4	10	10	30	40	–
	Grade	I		3 (+1)	2	6	3 (+1)	12 (+6)	22	*p* = 0.2
		II		1	1 (+1)	3	1	5 (+5)	11	
		III		0	1 (+2)	3	0	1 (+1)	2	
	Size	<1 cm (A)		0	0	0	4 (+3)	6 (+1)	14	***p*** < **0.0001**
		1–3 cm (A)		2 (+1)	1	4	12 (+4)	14 (+9)	39	
		3–5 cm (AB)		0 (+1)	1	2	0	5 (+3)	8	
		>5 cm (B)		6 (+1)	2 (+2)	11	1 (+1)	4 (+1)	7	

Different capital letters in the same column, within the same variable, indicate statistically significant differences. Bold values are statistically significant (*p* < 0.05).

Tumor size (>3 cm) (*p* = 0.02), diagnosis of anaplastic carcinoma (*p* = 0.0008) or grade II (*p* = 0.001)/III (*p* = 0.04) mammary gland tumors were associated with a higher probability of ALN metastasis. The superficial inguinal lymph node was more likely to have metastasis if there was increased tumor size (>5 cm) (*p* = 0.02) and in the presence of squamous cell carcinoma (*p* = 0.04).

## 4. Discussion

Data from research on women with breast cancer demonstrate that ALN metastasis are critical prognostic factors (16). The SLN biopsy is essential for a more precise staging, as tumor size, presence of lymph nodes and distant metastasis are all relevant prognostic factors in women as in canine patients (1, 17, 18).

Palpation, ultrasonography, and fine needle aspiration cytology provide some information on the lymph node status. However, as observed in this study, 80% (4/20) of lymph nodes with metastases were normal in size or not palpable. Nevertheless, the physical examination should not be neglected since the increased size and consistency of regional lymph nodes are associated with a higher risk of metastases, like previous studies in other tumour types (8, 19).

In veterinary medicine numerous techniques have been used to evaluate SLN (20). Although not common in veterinary medicine, some veterinarians already include the use of PB dye in evaluating the SLN. The technique for identifying lymph nodes with blue dyes is effective and presents low cost, with increased sensitivity. After 5–10 min of administration of a blue dye, it is already possible to identify the local lymphatic drainage and the corresponding SLN through the skin or below it after the incision (21). Our study showed a reduction in median surgical time from 80 to 45 min with the use of the PB dye. Furthermore, the subpapillary administration of PB resulted in 100% specificity and 92.6% sensitivity for identifying the ipsilateral ALN in female dogs with MGT, like what has been previously published (22). A recent study demonstrated the removal of ALN by a minimally invasive technique through endoscopy in dogs. In two patients, surgical times were 58 and 35 min, and one required conversion to an open approach. Despite the small sampling, the results indicated a promising technique (23).

Subpapillary administration is more straightforward than intradermal with a lower risk of subcutaneous administration which would stain all the subcutaneous tissue compromising lymph node identification. In this study, considering the low probability of contralateral drainage, the PB was administered subpapillary to identify the ipsilateral ALN (24, 25).

In our study, two patients did not have lymph nodes stained by PB, although they were still identified and removed. One such patient was a mixed-breed dog, with a flat tumor (2 cm), adhered to the skin and musculature, in the cranial thoracic mammary gland, diagnosed as an anaplastic carcinoma with lymphatic infiltration. The ALN was macroscopically abnormal (enlarged, firm and irregular), and had microscopically confirmed metastasis. It is essential to highlight that local lymphatic drainage can often be compromised by the presence of neoplastic emboli, as probably occurred in this dog.

In the present study, metastases in the ALNs were more frequent than in inguinal lymph nodes. However, the experimental design favored the selection of female dogs with neoplasms in the thoracic and abdominal cranial mammary glands, therefore, with axillary drainage. There is a paucity of information regarding the incidence of regional ALN metastasis in dogs with MGT, as ALN are often only removed when enlarged or with cytology-confirmed tumor metastasis. Furthermore, the rate of malignancy is variable among geographic areas. Higher rates of malignant MGT may occur in areas where pet owners have less access to veterinary care and present with cancer at more advanced stage.

An epidemiological study on the state of Espírito Santo, Brazil, with 255 dogs and 486 MGT, had 67% of malignancy. Only 106 patients had their lymph nodes evaluated and 53 had metastases (24.4%) (26). In our study, we found a similar percentage of malignant mammary carcinoma, among 87 lymph nodes evaluated by histopathology, 20 (23%) were metastatic.

None of the patients had any adverse reactions to the subpapillary administration of PB, as documented by Beserra et al. (27), but differently from Quadros and Gebrim (28) study in women, who reported 1% of adverse reactions, such as urticaria, in addition to trans and postoperative transient changes in oxygen saturation, with no clinical implication for the patient.

In this study, tumor location did not influence the occurrence of malignancy and histopathological grading. The probability of ALN metastasis was higher in patients with anaplastic carcinoma, an expected finding due to its aggressive biological behavior (1, 13). Other histopathological subtypes did not correlate with the occurrence of lymph node metastasis, but this could be due to type I error due to the low number of samples available for each histopathological subtype. However, lymph node metastasis was correlated with histopathological grading, with grade II or III tumors having a higher probability of lymph node metastasis.

This prospective study has some limitations. The number of cases was small to allow a multivariate statistical analysis, a larger number of samples might allow more robust conclusions. The samples were not randomized, and clinician selection bias of using PB versus not, cannot be ruled out. In dogs with no indication for resection of the inguinal mammary glands the presence of metastasis in the inguinal lymph node was not evaluated, however, drainage to this lymph node would be extremely unlikely. Immunohistochemistry was not carried out to investigate metastases in the lymph nodes considered negative, the cytokeratin stain might have increased the detection of metastases (29).

## 5. Conclusion

PB is helpful in ALN’s identification and reduces surgical time in dogs with MGT. In dogs with thoracic MGT, metastasis to the ALNs could occur more often than previously reported. ALN detection and removal is highly recommended especially in dogs presenting MGT larger than 3 cm or histopatologically diagnosed with an aggressive subtype or grade II or III.

## Data availability statement

The raw data supporting the conclusions of this article will be made available by the authors, without undue reservation.

## Ethics statement

The animal study was reviewed and approved by Ethics Committee on the Use of Animals from Universidade Vila Velha (Brazil), n° 377/2016.

## Author contributions

MS, MF, and RH contributed to conception, design of the study, and performed the study. FA performed the study. RH performed the statistical analysis. FA, BS, and AG wrote sections of the manuscript. All authors contributed to the article and approved the submitted version.

## Funding

This study was supported by Fundação de Amparo à Pesquisa e Inovação do Espírito Santo (FAPES).

## Conflict of interest

The authors declare that the research was conducted in the absence of any commercial or financial relationships that could be construed as a potential conflict of interest.

## Publisher’s note

All claims expressed in this article are solely those of the authors and do not necessarily represent those of their affiliated organizations, or those of the publisher, the editors and the reviewers. Any product that may be evaluated in this article, or claim that may be made by its manufacturer, is not guaranteed or endorsed by the publisher.

## Footnotes

^1^Cristália®.

^2^Agener União®.

^3^ABL Brasil®.
